# Health coaching for individuals with type 2 diabetes: assessing the impact of health coaching on HbA1c, hospitalizations, and outpatient services

**DOI:** 10.3389/fendo.2025.1443490

**Published:** 2025-09-17

**Authors:** Anthony Gittens, Ernie Medina, Jisoo Oh, Anna Nelson, Adam Aréchiga

**Affiliations:** School of Public Health, Loma Linda University, Loma Linda, CA, United States

**Keywords:** healthcare utilization, type 2 diabetes, health coaching, medicaid, HbA1c

## Abstract

**Introduction:**

The global prevalence of type 2 diabetes mellitus (T2DM) presents substantial public health challenges, particularly among Medicaid populations. Health coaching has emerged as a promising intervention to improve glycemic control and healthcare utilization.

**Methods:**

We conducted a retrospective pre-post secondary data analysis of 4,583 CalOptima Medicaid recipients with T2DM between March 2015 and August 2023. Patients who received health coaching (n = 3,777) were compared to those who declined (n = 806). Primary outcomes included HbA1c, hospitalizations, and outpatient visits.

**Results:**

The coached group experienced a significantly greater reduction in HbA1c (MD = -1.14, SD = 1.98) compared to the non-coached group (MD = -0.80, SD = 1.96; t(4581) = 4.51, p < .001). Ambulatory visits increased significantly among coached participants (p < .001), though hospitalizations showed no significant changes. Logistic regression indicated coached individuals had higher, though not statistically significant, odds of achieving normal HbA1c levels (OR = 1.19, 95% CI: 0.96–1.46).

**Discussion:**

Health coaching was associated with improved glycemic control and increased ambulatory care engagement among Medicaid patients with T2DM. These findings highlight the value of patient-centered interventions in chronic disease management within underserved populations.

## Introduction

The global challenge of type 2 diabetes mellitus (T2DM) poses significant concerns for both public health and socioeconomic stability, particularly affecting vulnerable populations (1). In the United States, the Medicaid population represents a unique cohort with distinct challenges in diabetes management. A complex interplay of socioeconomic determinants of health, such as access to quality care, health literacy, and social support systems, exacerbates these challenges.

Expanding upon the socio-ecological framework, it becomes evident that individual behavior change in T2DM management is influenced by factors at various levels—from personal beliefs to institutional policies (2). The intricate relationship between socioeconomic status and health outcomes necessitates interventions that are both personalized and systemic (3). Health coaching, situated within this framework, emerges as a promising multifaceted approach.

Literature suggests that while many health interventions for T2DM have successfully achieved short-term goals, maintaining improved health behaviors remains a challenge (4). Health coaching can potentially bridge this gap by providing ongoing support that is adaptable to the individual’s changing circumstances and health status (5). Moreover, previous interventions have highlighted the potential benefits of health coaching in improving glycemic control and reducing hospital admissions (6). However, a gap remains in understanding the full extent of its impact on the Medicaid population, particularly given the variance in program implementation and adherence rates (7). One intervention focused on goal-oriented collaborations between health professionals and patients, aiming to promote engagement in healthy lifestyle changes and self-management (8).

Health educators often struggle to reach individuals with lower educational backgrounds and socioeconomic status. Health coaching could go a long way in addressing the needs of this population, as it was initially developed in part to help individuals establish and attain goals to improve their health behaviors and overall health-related quality of life (9).

This study aimed to explore the impact of health coaching on the HbA1c levels, hospitalizations, and outpatient service utilization of Medicaid recipients with T2DM within Orange County’s CalOptima Population Health Management Program. Orange County’s CalOptima Population Health Management Program serves over 900,000 low-income residents, many of whom are diagnosed with T2DM. These individuals often face multiple challenges, including language barriers, limited transportation, food insecurity, and low health literacy levels. Previous initiatives, such as Project Dulce and the Medicaid Health Home initiative, have successfully improved HbA1c levels and reduced hospitalizations through culturally tailored, team-based approaches. However, there is limited evidence specifically examining the impact of health coaching within a large-scale Medicaid-managed care environment, such as CalOptima. Despite the potential benefits of health coaching, its implementation in diabetes care—particularly within underserved populations—has faced considerable challenges. These include inconsistent program fidelity, lack of patient engagement, and variability in coach training. Some institutions have struggled to.

## Materials and methods

### Data and participants

The present study employed secondary data analysis using a pre-post design to assess the effects of health coaching on three outcomes: HbA1c levels, the number of hospitalizations, and the number of outpatient care visits.

To determine the effectiveness of the CalOptima health coaching program, our study used Healthcare Effectiveness Data and Information Set (HEDIS) measures. HEDIS is a tool used by over 90% of health plans in America to measure the quality of care and service of an organization (10). We included Medicaid recipients over 18 with a confirmed diagnosis of T2DM who resided in Orange County and were enrolled in the CalOptima Population Health Management Program. Participants who consented could fully engage in the health coaching program for six months. All participants had adequate electronic medical records that were retrospectively reviewed.

The analysis was reviewed and approved as exempt by the Loma Linda University Institutional Review Board. Per ethical standards, data were collected in compliance with HIPAA. Participants consented at the time of their CalOptima enrollment to the use of their de-identified data for quality improvement and research.

Data for all dependent variables in this study (1) HbA1c, (2) number of hospitalizations, and (3) outpatient care services were collected from an electronic record for each patient. These variables are also among the HEDIS measurements that CalOptima reports to the National Committee for Quality Assurance biannually. Data collection included HbA1c measurements 9 months before health coaching and 3 to 12 months after coaching. Hospitalization data were collected to reflect inpatient admissions related to diabetes complications, encompassing a range of conditions from hypoglycemia to diabetic amyotrophy. The utilization of ambulatory care services and hospitalization data was compared for the 12 months preceding and 3 to 12 months following the health coaching intervention. Age, sex, and race/ethnicity were included as covariates. We created a category for Vietnamese because they comprised a large part of the sample (15.4%), similar to the White category (15.7%) of the sample.

The health coaching intervention consisted of monthly telephonic sessions typically spanning six months. The initial session usually lasted about one hour, with subsequent sessions averaging 30 minutes. The CalOptima health coaching program employed a client-centered educational approach. Coaches supported participants in developing self-management strategies for diabetes care, including medication adherence, recognizing symptoms of blood sugar fluctuations, and identifying when to seek urgent care or contact their healthcare professionals.

Health coaches selected for this program had a minimum of two years of prior experience in health coaching. They held a minimum of a bachelor’s degree in health science, nursing, nutrition, public health, social work, or other related fields. Health coaches with clinical backgrounds held unrestricted licenses as registered nurses (RNs), certified physician assistants (PAs), or registered dietitians in California. Moreover, many held additional certifications, including Certified Case Manager (CCM), Chronic Care Professional (CCP), Certified Diabetes Care and Education Specialist (CDCES), Certified Health Education Specialist (CHES), and Asthma Educator (AE-C). While not mandatory, having certified health coach credentials was recommended. Newly recruited health coaches underwent an intensive, month-long orientation to refine their skills in active listening, goal setting, providing constructive feedback, mirroring, and trust-building — essential skills for effective health coaching.

### Variables and measurements

*Independent Variable.* The independent variable in this study was the CalOptima Health Coaching Program.

*Dependent Variables.* Our study assessed three dependent variables: HbA1c, number of diabetes-related hospitalizations, and number of outpatient services used.

*HbA1c.* Blood sugar measurements (HbA1c mg/dL%) were recorded for all participants within 9 months before the start of health coaching or when health coaching was declined and within 3 to 12 months after.

*Number of Hospitalizations.* The number of inpatient stays was also assessed in this study. These types of visits were characterized by severe injury or sudden illness that required immediate attention.

*Number of Outpatient Visits.* Outpatient visits (ambulatory visits) are a measure that includes outpatient (non-emergency) visits, emergency room visits, telephone consultations, e-visits, and virtual check-ins.

### Analytical strategy

For this research, we removed 281 outliers. The cutoffs for the outliers were applied by removing records where hospitalizations exceeded 20, ambulatory visits exceeded 40, and emergency visits exceeded 10. This yielded a sample size of 4,583 patients, comprising individuals who received health coaching (n = 3,777) and a comparative group (n = 806) that declined the coaching.

Using Stata SE v15, a paired t-test comparing pre-and post-coaching HbA1c levels and number of patient visits, an independent samples t-test comparing coached versus non-coached patients on post minus pre-coaching differences in HbA1c levels and number of patient visits, and a binary logistic regression model to predict normal (≤ 6.5%) post-coaching HbA1c levels were used to assess the impact of health coaching on changes in HbA1c levels, number of hospitalizations, and number of outpatient care visits.

Descriptive statistics were calculated for all study variables. The normality of the primary outcome variable (HbA1c) was assessed using the Shapiro-Wilk test prior to performing inferential statistical tests. Paired t-tests were used to evaluate changes in HbA1c, hospitalizations, and outpatient visits pre- and post-coaching. Independent samples t-tests were used to compare changes between the coached and non-coached groups. Binary logistic regression was conducted to predict the likelihood of achieving HbA1c <6.5%.

## Results

**Table 1** provides the demographic characteristics of the study sample. First, it is essential to note that most participants (82.4%) accepted coaching, while 17.6% declined the program. Females comprised the majority (~57.5%) of the sample. About 84.8% of females accepted health coaching. Among males, 79.2% received coaching, while 20.8% did not. The Chi-square (*χ*2) indicated a significant difference between coached and non-coached groups in terms of gender. Approximately 59.1% of the sample identified as Hispanic, 15.7% as White, and 15.4% as Vietnamese. Notably, the percentage of Black individuals within the study sample was relatively small at 1.8%. Approximately 81.1% of Hispanics accepted health coaching. Among White patients, 77.2% received coaching, and 75.6% of Black patients received coaching. The Vietnamese group showed the highest coaching rate at 95.9%. Other Asian/Pacific Islander patients had 78.1% coached.

**Table 1:** Demographic comparisons between coached versus non-coached patients.

Variable	Category	Coached (*n* = 3777)		Non-coached (*n* = 806)		*χ2*	*p*
		*n*	*%*	*n*	*%*		
Gender	Female	2233	84.8%	401	15.2%	23.86	<.001
	Male	1544	79.2%	405	20.8%		
Race/Ethnicity (each *vs*. all others)							
	Hispanic	2198	81.1%	513	18.9%	8.18	.004
	White	554	77.2%	164	22.8%	16.22	<.001
	Black	62	75.6%	20	24.4%	2.67	.102
	Vietnamese	677	95.9%	29	4.1%	104.62	<.001
	Other Asian/Pacific Islander	286	78.1%	80	21.9%	5.01	.025
Age (linear trend test)							
	18-39 years	380	83.2%	77	16.8%	2.25	.134
	40-64 years	2854	82.8%	594	17.2%		
	65 years and older	543	80.1%	135	19.9%		

**Table 2** shows a general improvement in HbA1c levels following health coaching, indicating better blood glucose management. There was a slight decrease in emergency room and inpatient visits, but a slight increase in ambulatory visits occurred post-coaching. Similarly, for diabetes-related visits, there was a slight increase in emergency room visits, a minor decrease in inpatient visits, and an increase in ambulatory visits post-coaching.

**Table 2:** Descriptive statistics prior to and after health coaching for diabetes-related and all visits.

Variables	Pre-coaching			Post-coaching		
	*Mean*	*SD*	*Range*	*Mean*	*SD*	*Range*
HbA1c	9.18	1.90	4.6 - 17.4	8.10	1.78	4.4 - 16
All visits						
Emergency Room	0.79	1.38	0 - 10	0.75	1.40	0 - 10
Inpatient	0.41	1.38	0 - 20	0.34	1.21	0 - 19
Ambulatory	11.30	7.92	0 - 40	11.83	8.34	0 - 40
Diabetes-related visits						
Emergency Room	0.56	1.10	0 - 9	0.58	1.17	0 - 10
Inpatient	0.30	1.12	0 - 19	0.29	1.09	0 - 19
Ambulatory	5.65	4.60	0 - 35	6.20	4.82	0 - 37

**Table 3** presents the results of paired t-tests comparing HbA1c levels and the number of patient visits before and after health coaching. The mean HbA1c level decreased by 1.08 mg/dl after coaching (p<.001). Inpatient visits experienced a mean decrease of 0.07 (p = .004) across all types of visits. Ambulatory visits increased by 0.53 visits (p<.001). For diabetes-related visits, ambulatory visits increased by 0.56 visits (p<.001).

**Table 3:** Paired t-tests comparing pre-and post-coaching HbA1c levels and number of patient visits.

Variables	Paired differences				
	*Mean*	*SD*	*95% CI*	*Paired t (4582)*	*p*
HbA1c	-1.08	1.98	-1.14 to -1.02	-36.89	<.001
All visits					
Emergency Room	-0.04	1.53	-0.09 to 0.00	-1.92	.055
Inpatient	-0.07	1.62	-0.12 to -0.02	-2.89	.004
Ambulatory	0.53	7.75	0.31 to 0.76	4.66	<.001
Diabetes-related visits					
Emergency Room	0.02	1.28	-0.02 to 0.05	0.91	.362
Inpatient	-0.01	1.37	-0.05 to 0.03	-0.51	.613
Ambulatory	0.56	4.77	0.42 to 0.69	7.88	<.001

**Table 4** shows the differences in health outcomes between patients who received coaching and those who did not. For HbA1c levels, patients who received coaching experienced a significant improvement in their blood glucose control. The coached group had a mean difference of -1.14 with a standard deviation (SD) of 1.98, compared to a mean difference of -0.80 with an SD of 1.96 in the non-coached group. This difference was statistically significant (t(4581) = 4.51, p <.001). Furthermore, the coached group had a mean increase of 0.75 for all ambulatory visits compared to a decrease of 0.49 in the non-coached group (p<.001). For diabetes-related ambulatory visits, the coached group experienced a mean increase of 0.68 visits, compared to a decrease of 0.02 in the non-coached group (p <.001).

**Table 4:** Independent samples t-tests comparing coached versus non-coached patients on post minus pre-coaching differences in HbA1c levels and number of patient visits.

Variables	Coached (*n* = 3777)		Non-coached (*n* = 806)		*t (4581)*	*p*
	*Mean difference*	*SD*	*Mean difference*	*SD*		
HbA1c	-1.14	1.98	-0.80	1.96	4.51	<.001
All visits						
Emergency Room	-0.03	1.54	-0.12	1.49	-1.60	.110
Inpatient	-0.06	1.62	-0.13	1.59	-1.13	.258
Ambulatory	0.75	7.75	-0.49	7.63	-4.13	<.001
Diabetes-related visits						
Emergency Room	0.03	1.29	-0.04	1.23	-1.45	.146
Inpatient	0.00	1.36	-0.06	1.41	-1.07	.286
Ambulatory	0.68	4.80	-0.02	4.60	-3.75	<.001

**Table 5** presents the results of a binary logistic regression analysis to predict the likelihood of achieving normal post-coaching HbA1c levels (≤ 6.5%). This table demonstrated that coached individuals had 1.19 times higher odds (OR = 1.19, 95% CI: 0.96-1.46, p = .107) of achieving normal HbA1c levels compared to non-coached individuals. Hispanics were significantly less likely to achieve normal HbA1c levels compared to Whites, with an OR of 0.57 (95% CI: 0.46-0.69, p <.001). Similarly, Vietnamese individuals (OR: 0.71, 95% CI: 0.55-0.92, p = .009) and Other Asian/Pacific Islanders (OR: 0.67, 95% CI: 0.49-0.91, p = 0.011) were also less likely to achieve normal HbA1c levels compared to Whites. Individuals aged 40-64 have an OR of 0.69 (95% CI: 0.55-0.88, p = 0.002), and those aged 65 and older have an OR of 0.73 (95% CI: 0.54-0.97, p = .032) compared to the 18-39 age group, indicating that older age groups are less likely to achieve normal HbA1c levels.

**Table 5:** Binary logistic regression to predict normal (≤ 6.5%) post-coaching HbA1c levels.

Variable	*OR*	*95% CI*	*p*
Coaching			
Not Coached	*ref*	-	-
Coached	1.19	0.96 - 1.46	.107
Gender			
Female	*ref*	-	-
Male	0.90	0.77 - 1.05	.168
Race/Ethnicity			
White	*ref*	-	-
Hispanic	0.57	0.46 - 0.69	<.001
Black	0.82	0.47 - 1.43	.487
Vietnamese	0.71	0.55 - 0.92	.009
Other Asian/Pacific Islander	0.67	0.49 - 0.91	.011
Age			
18-39	*ref*	-	-
40-64	0.69	0.55 - 0.88	.002
65+	0.73	0.54 - 0.97	.032

It is important to note that HbA1c <6.5% was selected as the cutoff point in alignment with American Diabetes Association (ADA) guidelines for the diagnosis of diabetes. While this threshold is typically used for diagnosis rather than control, it serves as a benchmark for identifying patients achieving normoglycemia post-intervention.

## Discussion

The findings from our study demonstrated that health coaching had a positive impact on reducing HbA1c levels, indicating improved diabetes management. There were slight changes in the number of hospitalizations. Still, the most significant changes were the reduction in HbA1c levels and the increase in ambulatory visits following coaching, suggesting a potential shift towards more frequent routine care.

Previous work has consistently reported the positive effects of health coaching on HbA1c levels (11), reinforcing the findings of our study. Our results align with numerous studies that highlight the effectiveness of health coaching in managing T2DM. For instance, a systematic review and meta-analysis highlighted that health coaching interventions incorporating behavior change techniques can significantly reduce HbA1c levels, typically ranging from 0.2% to 0.4% (12). In comparison, our study demonstrated a more substantial reduction in HbA1c levels, with the coached group experiencing a significantly greater reduction than the non-coached group. Specifically, the coached group had a 12.42% decrease in HbA1c levels compared to an 8.71% decrease in the non-coached group. This larger reduction observed in our study may be attributed to the comprehensive nature of the coaching program and its tailored approach to the Medicaid population.

Our study revealed a significant increase in ambulatory visits among the coached group compared to the non-coached group. The increased utilization of ambulatory care services in the coached group can be attributed to several factors. Health coaching typically includes personalized follow-up plans, regular check-ins, and goal setting, encouraging patients to adhere to their scheduled visits. Additionally, coaching helps address barriers to accessing ambulatory care, such as transportation issues and scheduling conflicts, by providing tailored solutions and support (13).

In a pilot study examining the impact of Healthy at Home, a 12-week phone and Short Message Service-based digital health coaching program designed to lower blood sugar levels, health coaching was compared to usual diabetic care in a family medicine residency clinic through a randomized controlled trial (14). The study found that digital health coaching significantly improved participants’ HbA1c levels compared with the usual care group. These findings further validate the role of health coaching as an effective intervention for improving glycemic control among individuals with T2DM, highlighting the potential for even more significant improvements when interventions are well-structured and targeted to meet specific population needs.

Our study showed that Hispanics, Vietnamese, and other Asian/Pacific Islander groups were significantly less likely to achieve normal HbA1c levels compared to White individuals after health coaching. Communication inequalities play a significant role. Language barriers and cultural differences, which often necessitate the use of interpreters, can impede effective communication between healthcare providers and patients, leading to misunderstandings and lower adherence to health recommendations (15). In our study, we sometimes used interpreter services for specific languages, and cultural differences may have further complicated the communication process. These challenges underscore the need for culturally competent care, as highlighted by the American Diabetes Association’s report on health disparities in diabetes care (25). Studies have shown that culturally tailored interventions are more effective in improving health outcomes among minority populations. However, many health coaching programs may not be adequately adapted to meet the specific needs of diverse ethnic groups, thereby limiting their effectiveness (16).

Our study found that adults aged 40-64 have 31% lower odds of achieving normal HbA1c levels than those aged 18-39. This finding aligns with recent research indicating that middle-aged adults often face unique challenges in managing diabetes effectively. Factors such as increased responsibilities, higher stress levels, and the onset of age-related metabolic changes can complicate diabetes management in this age group (17). For adults aged 65 years and older, the odds of achieving normal HbA1c levels were 27% lower compared to adults aged 18-39 years. Older adults often experience greater difficulty in maintaining glycemic control due to factors such as polypharmacy, cognitive decline, and multiple comorbidities (18). The American Diabetes Association (ADA) emphasizes the importance of individualized treatment goals for older adults, balancing the benefits of glycemic control with the risks of hypoglycemia and other adverse effects (19). This necessitates individualized treatment goals that balance the benefits of glycemic control with the potential risks associated with it. These findings further underscore the necessity for tailored approaches in diabetes care for older populations, ensuring that interventions are designed to address the specific needs and challenges older adults face in managing their diabetes.

A previous study found that patients with chronic conditions, including T2DM, who received health coaching had a significantly better experience with their primary care. These patients reported a higher quality of care, attributed to the increased interaction with healthcare providers facilitated by health coaching. The study suggests that this enhanced interaction likely leads to more frequent ambulatory visits, thereby contributing to improved chronic disease management (20).

A systematic review and meta-analysis also examined the health coaching outcomes for individuals with T2DM (21). This review analyzed multiple studies and found that patients in the coached group had increased clinic visits and specialist consultations compared to the control group. This increase in healthcare utilization indicates a more proactive approach to health management facilitated by health coaching. By encouraging regular follow-ups and specialist consultations, health coaching helps patients better manage their diabetes, improving health outcomes and potentially reducing the incidence of acute complications (22).

A study evaluating the impact of pharmacist-led ambulatory care on patients with diabetes found that those receiving structured support showed significant improvements in glycemic control and increased use of ambulatory services. This highlights the effectiveness of multidisciplinary and patient-centered approaches in enhancing healthcare utilization (23). The consistent increase in ambulatory visits observed in various studies highlights the crucial role of health coaching in promoting sustained engagement with healthcare services, which is essential for the effective management of chronic diseases.

Our study highlighted significant gender differences in the effectiveness of health coaching for managing HbA1c levels, with females being more likely to be in the coached group than males. This finding aligns with existing literature that underscores the importance of gender-specific approaches in health interventions. Research indicates that women are generally more proactive in seeking healthcare and participating in health programs, which may explain their higher representation in the coached group (24).

### Strengths

Our study exhibits several notable strengths that distinguish it from other research in the field. The large sample size of 4,583 respondents provides a robust dataset, enhancing the reliability and generalizability of the findings. This large and diverse sample, focusing on Medicaid recipients with T2DM in Orange County’s CalOptima Population Health Management Program, ensures that the results are more representative and applicable to various subgroups within the Medicaid population.

Our study also provided a detailed analysis of demographic factors, including race, ethnicity, and age. This granularity helps to identify specific subgroups that may benefit more from health coaching, ensuring that interventions can be more precisely targeted and effective. For example, our study found that Hispanic patients and Asians were less likely to achieve target HbA1c levels compared to White patients, highlighting the need for culturally sensitive interventions.

### Limitations

Although this study offers valuable insights into the impact of health coaching, it is essential to acknowledge its limitations. Firstly, we lacked data on crucial clinical and contextual variables, such as the type of pharmacological treatment, diabetes-related complications (e.g., neuropathy, retinopathy), the duration of the diagnosis, and patient comorbidities. These factors may influence both the effectiveness of coaching and patient outcomes. Secondly, there may have been selection bias, as patients who chose to participate in coaching might differ systematically from those who declined.

Additionally, the study’s inability to control for other confounding variables may present a limitation. Confounding variables, such as socioeconomic status, comorbid conditions, and access to healthcare resources, may impact health outcomes and the effectiveness of interventions. Without access to and control over these variables, it is challenging to isolate the specific effects of health coaching on ambulatory care visits and HbA1c levels. This lack of control means that other unmeasured factors could have influenced the results, potentially biasing the findings. Future studies should incorporate more comprehensive clinical datasets, consider longitudinal designs, and a broader range of confounding variables to provide a more comprehensive understanding of the impact of health coaching.

### Implications for practice

Our study may help healthcare professionals working with patients with T2DM by comparing the outcomes of outpatient visits before and after receiving health coaching as part of their health management plan. Healthcare practitioners and patients may recognize the importance of health coaching programs in promoting self-management skills that can help address health complications, including early detection, prevention, and treatment, thereby reducing the likelihood of further medical complications.

Our research findings provided insight into how CalOptima and similar healthcare programs could improve patients’ hemoglobin A1C levels and ambulatory care services. This information can help healthcare facilities in their quest to create and implement various programs for enhanced patient care, whereby patients with T2DM can be supported through improved approaches to control hemoglobin A1C levels and through improved access to ambulatory care services.

Health coaching is a patient-oriented practice that aims to motivate individuals to change their behavior. Administrators of Medicaid facilities may find our study’s findings helpful, as they understand that health coaching enables the coach to provide support, education, and feedback within a client-centered context, thereby enhancing patients’ self-management skills. This approach allows for the client to experience enhanced self-awareness, motivation, accountability, and self-efficacy, while also acknowledging that patients are experts in their own life situations and should be the ones to provide direction for learning and change (16).

### Recommendations for future research

Further research may be conducted using data from other research settings, including different healthcare facilities in Orange County and other counties and states. Future researchers may consider adopting qualitative research methods by including patients with T2DM and health coaches to gather detailed information about health coaching and its impact on patients’ HbA1c levels.

Another recommendation for future research involves delving deeper into the coaching programs offered and analyzing their components. Although participation in coaching was treated as an indicator variable where the data only indicated whether training occurred or not, improvements in T2DM outcome and HbA1c levels are likely a result of what participants learn during training and how they apply the training content to their treatment regimen. Future studies could partition health coaching into multiple domains to analyze which aspect of health coaching is the most beneficial in improving patients’ HbA1c levels.

## Concluding remarks

This study was prompted by the continued increase in the global prevalence of T2DM as one of the primary causes of morbidity and mortality, along with its substantial personal and economic burden. Health coaching aims to empower patients with T2DM to manage their condition using various self-management skills effectively. It aims to motivate patients to achieve their goals, thereby enhancing their quality of life and other health-related domains. This study offers insights into how the CalOptima health coaching program impacts the utilization of outpatient services among Medicare patients diagnosed with T2DM.

Our study has provided important information about the role of health coaching in outpatient service utilization and HbA1c levels. Health coaching interventions can help improve T2DM by promoting healthy lifestyle changes and self-management. Patients and healthcare professionals may benefit from this study in understanding the importance of factors such as age and ethnicity in predicting HbA1c levels. Most importantly, healthcare professionals and individuals diagnosed with T2DM may realize the need for health coaching as an intervention for diabetic health conditions.

## Data Availability

The raw data supporting the conclusions of this article will be made available by the authors, without undue reservation.
